# Growth‐Correcting the Bioconcentration Factor and Biomagnification Factor in Bioaccumulation Assessments

**DOI:** 10.1002/etc.4509

**Published:** 2019-08-01

**Authors:** Frank A.P.C. Gobas, Yung‐Shan Lee

**Affiliations:** ^1^ School of Resource and Environmental Management Simon Fraser University Burnaby British Columbia Canada

**Keywords:** Bioaccumulation, Assessment, Bioconcentration, Biomagnification, Hydrophobic chemicals

## Abstract

We illustrate that the Organisation for Economic Co‐operation and Development guideline 305 (OECD‐305) for growth‐correcting bioconcentration factors (BCFs) and biomagnification factors (BMFs) violates the mass‐balance assumption underlying the definition of BCFs and BMFs and provides unrealistic estimates of BCFs and BMFs of chemicals in nongrowing fish. We present and test alternative methods for growth‐correcting BCFs and BMFs that maintain mass balance. We conclude that the OECD‐305‐recommended growth correction of BCFs and BMFs causes error, is unnecessary, and should be revisited. *Environ Toxicol Chem* 2019;38:2065–2072. © 2019 The Authors. *Environmental Toxicology and Chemistry* published by Wiley Periodicals, Inc. on behalf of SETAC.

## Graphical abstract

Effect of growth on bioconcentration factors and biomagnification factors.

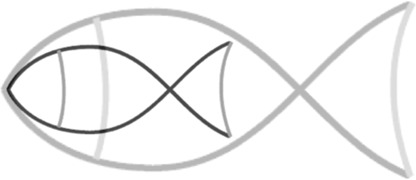

## INTRODUCTION

The guidelines developed by the Organisation for Economic Co‐operation and Development (OECD) for conducting aqueous and dietary bioaccumulation tests (guideline 305; Organisation for Economic Co‐operation and Development 2012) include methods for determining growth‐corrected bioconcentration factors (BCFs) and biomagnification factors (BMFs), meant to represent the BCFs and BMFs of chemicals in nongrowing fish. The reason for the growth correction is that in most bioaccumulation tests fish are provided with sufficient food to sustain a healthy growth rate, while in natural environments fish may encounter periods with limited access to food, causing growth rates of fish to be smaller than those in bioaccumulation tests. The growth correction involves subtracting the growth rate of the fish measured in the experiment and expressed in terms of the specific growth rate or growth dilution rate constant (*k*_g_) from the measured depuration rate constant (*k*_T_) of the chemical in the fish. The difference (i.e., *k*_T_ – *k*_g_) is then used to calculate the kinetic BCF_NG_ and BMF_NG_ of the test chemical in a nongrowing (NG) fish as

(1) $${\text{BCF}}_{\text{NG}}=\frac{k_{1}}{\left(k_{T}-k_{g}\right)}\;\text{and}\;{\text{BMF}}_{\text{NG}}=\frac{E_{D}\;\cdot \;F_{D}}{\left(k_{T}-k_{g}\right)}$$

where *k*_1_ is the clearance rate for respiratory uptake (L/kg fish per day), *E*_*D*_ is the dietary uptake efficiency (unitless), *F*_*D*_ is the feeding rate (kilograms of food per kilogram of fish per day), *k*_T_ is the rate constant for depuration (per day), and *k*_g_ is the growth dilution rate constant (per day). The BCF_NG_ and BMF_NG_ are greater than the actually measured kinetic BCF and BMF in the experiment and under certain circumstances (e.g., fast‐growing experimental fish) can become very much greater than the measured BCF and BMF.

Although there are good reasons to conduct bioaccumulation assessments for slowly or nongrowing fish, the method for assessing the BCF and BMF in nongrowing fish from bioaccumulation test results with growing fish needs to be revisited because the current method described in the OECD‐305 guideline violates the mass‐balance assumption, on which the bioaccumulation model and the correct determination of the BCF and BMF are based. The violation of mass balance in the growth correction occurs when, by subtracting *k*_g_ from *k*_T_, the loss of chemical mass from the fish is reduced whereas the intake of chemical mass is not. This causes the numerator in the BCF and BMF to be represented by a growing fish and the denominator by a nongrowing fish. This is not a realistic description of bioaccumulation in a nongrowing fish and the corresponding BCFs and BMFs do not correctly represent the bioaccumulation of the chemical in nongrowing fish.

It is well recognized that, under normal conditions, growing fish exhibit a higher feeding rate than the same nongrowing fish (e.g., Kiørboe et al. 1987; Kiørboe and Møhlenberg 1987). It is therefore not correct to use the feeding rate of growing fish in the test to calculate the BMF in nongrowing fish. This can be confirmed by investigating the relationship between the fish's feeding rate, which is specified in the bioaccumulation test, and the fish's growth rate, which is routinely measured.

It is also recognized that feeding is associated with an increase in oxygen consumption rate in a range of fish species (Muir and Niimi 1972; Hamada and Ida 1973; Beamish 1974; Brett 1976; Miura et al. 1976; Tandler and Beamish 1979) and that oxygen consumption rates in fish increase with the specific growth rate of the fish (Jobling 1981), which is the same as the growth dilution rate constant determined in bioaccumulation tests. It is therefore also not correct to use the respiration rate of growing fish in bioaccumulation tests to represent the respiration rate in nongrowing fish. Unfortunately, the relationship between oxygen consumption and growth rates cannot easily be determined from the results of bioaccumulation tests because oxygen consumption rates and associated gill ventilation rates are not routinely measured in bioaccumulation tests. However, information on the relationship between oxygen consumption and growth rates can be obtained from relevant studies of the physiology of respiration in fish.

The objectives of the present study were as follows. First, we aimed to develop a theoretical framework for assessing the BCF and BMF of a chemical in nongrowing fish from the results of bioaccumulation tests with growing fish that maintains mass balance. Second, we applied the framework to assess the BCF and BMF of a chemical in nongrowing fish and compared the results to those derived from using the OECD‐305‐recommended method for growth‐correcting the BCF and BMF. Third, we investigated the relationship between the feeding rate and the growth rate in fish of dietary bioaccumulation tests to test the methods for growth correction of the BMF. Fourth, we explored the relationship between oxygen consumption and the specific growth rate in fish to test the method for growth correction of the BCF. Finally, we explored the magnitude of error caused by the OECD‐305 growth correction and its significance for bioaccumulation assessments. The ultimate goal of the present study was to improve methods for bioaccumulation assessment of chemicals in fish.

## THEORY

Following the fish–water and fish–food 2‐compartment models, used in the OECD‐305 guidelines (Organisation for Economic Co‐operation and Development 2012) to describe the bioaccumulation of chemicals in fish from water and food, the mass‐balance equations for a test chemical in fish of an OECD‐305 bioaccumulation test can be described as

(2) $$\frac{d(W_{F}\;\cdot \;C_{F})}{dt}=C_{F}\frac{dW_{F}}{dt}+W_{F}\frac{dC_{F}}{dt}=k_{1}^{*}\;\cdot \;V_{W}\;\cdot \;C_{W}-k_{E}\;\cdot \;W_{F}\;\cdot \;C_{F}$$

in aqueous bioconcentration tests and as

(3) $$\frac{d(W_{F}\;\cdot \;C_{F})}{dt}=C_{F}\frac{dW_{F}}{dt}+W_{F}\frac{dC_{F}}{dt}=E_{D}\;\cdot \;F_{D}^{*}\;\cdot \;C_{D}-k_{E}\;\cdot \;W_{F}\;\cdot \;C_{F}$$

in dietary bioaccumulation tests, where *C*_*F*_, *C*_*W*_, and *C*_*D*_ are the concentrations of the chemical in, respectively, the body of the fish (moles per kilogram of fish), water (moles per liter), and the food of the fish (moles per kilogram of food); *k*_1_* is the rate constant for respiratory uptake (per day); *E*_*D*_ is the dietary uptake efficiency (unitless); *F*_D_* is the feeding rate (kilograms of food per day); *k*_E_ is the rate constant for depuration (per day), which combines all depuration routes including gill elimination, biotransformation, and fecal excretion (but not growth dilution because the chemical is not leaving the fish); *W*_*F*_ is the weight of the fish (kilograms) and *V*_*W*_ is the volume of water (liters) that the fish is exposed to.

For a nongrowing fish, $\frac{dW_{F}}{\mathrm{dt}}=0$ and on division by *W*_*F*_ Equations (1) and (2) become

(4) $$\frac{dC_{F}}{dt}=k_{1}^{*}\;\cdot \;\left(\frac{V_{W}}{W_{F}}\right)\;\cdot \;C_{W}-k_{E}\;\cdot \;C_{F}=k_{1}\;\cdot \;C_{W}-k_{E}\;\cdot \;C_{F}$$

and

(5) $$\frac{dC_{F}}{dt}=E_{D}\;\cdot \;F_{D}^{*}\;\cdot \;\left(\frac{1}{W_{F}}\right)\;\cdot \;C_{D}-k_{E}\;\cdot \;C_{F}=E_{D}\;\cdot \;F_{D}\;\cdot \;C_{D}-k_{E}\;\cdot \;C_{F}$$

where *F*_*D*_ is the proportional feeding rate (kilograms of food per kilogram of fish per day) and *k*_1_ is the respiratory clearance rate (liters per kilogram of fish per day). The BCF_NG_ and BMF_NG_ in a nongrowing fish are defined at steady state (i.e., $\frac{dC_{F}}{\mathrm{dt}}=0$) as

(6) $${\text{BCF}}_{\text{NG}}=\frac{C_{F}}{C_{W}}=\frac{k_{1}}{k_{E}}\;\text{and}\;{\text{BMF}}_{\text{NG}}=\frac{C_{F}}{C_{D}}=\frac{E_{D}\;\cdot \;F_{D}}{k_{E}}$$

In experiments in which fish are growing, $\frac{dW_{F}}{\mathrm{dt}}\neq 0$. Subtracting $C_{F}\frac{dW_{F}}{\mathrm{dt}}$ on both sides of Equations (2) and (3), dividing by *W*_*F*_ and assuming that $\frac{dW_{F}}{W_{F}\mathrm{dt}}$ is constant (i.e., the growth rate constant, *k*_g_) then transforms Equations (2) and (3) into

(7) $$\frac{dC_{F}}{dt}=k_{1}^{*}\;\cdot \;\left(\frac{V_{W}}{W_{F}}\right)\;\cdot \;C_{W}-k_{E}\;\cdot \;C_{F}-\frac{dW_{F}}{W_{F}dt}\;\cdot \;C_{F}\;=k_{1}\;\cdot \;C_{W}-k_{E}\;\cdot \;C_{F}-k_{g}\;\cdot \;C_{F}$$

and

(8) $$\frac{dC_{F}}{dt}=E_{D}\;\cdot \;F_{D}^{*}\;\cdot \;\left(\frac{1}{W_{F}}\right)\;\cdot \;C_{D}-k_{E}\;\cdot \;C_{F}-\frac{dW_{F}}{W_{F}dt}\;\cdot \;C_{F}\;=E_{D}\;\cdot \;F_{D}\;\cdot \;C_{D}-k_{E}\;\cdot \;C_{F}-k_{g}\;\cdot \;C_{F}$$

At steady state (i.e., $\frac{dC_{F}}{\mathrm{dt}}=0$), the mass‐balance equations for a chemical in a growing fish are

(9) $$k_{1}\;\cdot \;C_{W}=k_{E}\;\cdot \;C_{F}+k_{g}\;\cdot \;C_{F}=k_{T}\;\cdot \;C_{F}$$

and

(10) $$E_{D}\;\cdot \;F_{D}\;\cdot \;C_{D}=k_{E}\;\cdot \;C_{F}+k_{g}\;\cdot \;C_{F}=k_{T}\;\cdot \;C_{F}$$

where the total depuration rate constant, *k*_T_ (per day) is the sum of *k*_E_ and *k*_g_ (i.e., *k*_T_ = *k*_E_ + *k*_g_). The BCF and BMF in a growing fish are

(11) $$\text{BCF}=\frac{C_{F}}{C_{W}}=\frac{k_{1}}{k_{E}+k_{g}}=\frac{k_{1}}{k_{T}}\;\text{and}\;\text{BMF}=\frac{C_{F}}{C_{D}}=\frac{E_{D}\;\cdot \;F_{D}}{k_{E}+k_{g}}=\frac{E_{D}\;\cdot \;F_{D}}{k_{T}}$$

In OECD‐305 bioaccumulation tests, the total depuration rate constant (*k*_T_) is typically determined from the slope of a linear regression of ln *C*_*F*_ and time and represents the sum of *k*_E_ and *k*_g_. The growth correction involves subtracting *k*_g_ from *k*_T_, giving *k*_E_, and calculating the BCF and BMF as *k*_1_/*k*_E_ and E_D_ · *F*_*D*_/*k*_E_, respectively, which appear to resemble Equation (6) for a nongrowing fish. This growth correction, however, is not correct. It violates the mass‐balance assumption underlying the correct determination of the BCF and BMF by subtracting *k*_g_ · *C*_*F*_ from the right side of Equations (9) and (10) but not the left side. This violation of mass balance would be acceptable if the feeding and respiratory rates are independent of the growth rate, but this is not the case because growing fish eat more than nongrowing fish (all else being equal; Kiørboe et al. 1987) and respire more water than nongrowing fish (Jobling 1981). The impact of the violation of mass balance is an overestimation of the BCF and BMF because the calculation of the BCF and BMF uses the uptake rates of a growing fish (in the numerator) but the depuration kinetics of a nongrowing fish (in the denominator). The BCFs and BMFs that are derived using this method of growth correction should be regarded as suspicious because they have no basis in reality. The option in the OECD‐305 guideline to derive the growth‐corrected depuration rate constant from a linear regression of the chemical mass (instead of concentration) versus time is an adequate way to derive a growth‐corrected depuration rate constant but is subject to the same violation of mass balance when the depuration rate constant for the nongrowing fish is combined with the feeding or respiration rate of the growing fish to determine the BCF or BMF.

The violation of the mass balance can be remedied by subtracting *k*_g_ · *C*_*F*_ from both sides of Equations (9) and (10):

(12) $$k_{1}\;\cdot \;C_{W}-k_{g}\;\cdot \;C_{F}=k_{E}\;\cdot \;C_{F}$$

and

(13) $$E_{D}\;\cdot \;F_{D}\;\cdot \;C_{D}-k_{g}\;\cdot \;C_{F}=k_{E}\;\cdot \;C_{F}$$

The left side of Equations (12) and (13) now represent the intake of chemical mass per day that is not allocated to growth, whereas the right side of the equation represents depuration of chemical excluding the effect of growth dilution. This chemical intake can represent the chemical intake in a nongrowing fish and is expressed in terms of the respiratory clearance rate (*k*_1,NG_) or the feeding rate in a nongrowing fish (*F*_*D*,NG_). (See Supplemental Data for step‐by‐step derivation of Equations (14) and (15).)

(14) $$k_{1,\text{NG}}=(k_{1}\;\cdot \;C_{W}-k_{g}\;\cdot \;C_{F})/C_{W}=k_{1}\;\cdot \;(1-\frac{k_{g}}{k_{T}})$$

(15) $${F_{D,\text{NG}}\;=\;(E}_{D}\;\cdot \;F_{D}\;\cdot \;C_{D}-k_{g}\;\cdot \;C_{F})/{(E}_{D}\;\cdot \;C_{D})\;=\;F_{D}\;\cdot \;(1-\frac{k_{g}}{k_{T}})$$

The respiratory clearance rate (*k*_1,NG_) and the feeding rate in a nongrowing fish (*F*_*D*,NG_) can then be used in the calculation of the BCF_NG_ and BMF_NG_ of a nongrowing fish

(16) $$BCF_{\text{NG}}=\frac{k_{1,\text{NG}}}{k_{E}}=\frac{k_{1}}{k_{E}}\;\cdot \;\left(1-\frac{k_{g}}{k_{T}}\right)=\frac{k_{1}}{\left(k_{T}-k_{g}\right)}\;\cdot \;\left(1-\frac{k_{g}}{k_{T}}\right)$$

(17) $$BMF_{\text{NG}}=\frac{E_{D}\;\cdot \;F_{D,\text{NG}}}{k_{E}}=\frac{E_{D}\;\cdot \;F_{D}}{k_{E}}\;\cdot \;\left(1-\frac{k_{g}}{k_{T}}\right)\;=\frac{E_{D}\;\cdot \;F_{D}}{\left(k_{T}-k_{g}\right)}\;\cdot \;(1-\frac{k_{g}}{k_{T}})$$

The term $\left(1-\frac{k_{g}}{k_{T}}\right)$ can be viewed as the reduction in the feeding rate or respiration rate that is associated with a lack of growth. Hence, it is possible to derive the BCFs and BMFs of chemicals in a nongrowing fish from the experimental results of a test with growing fish without violating the mass‐balance assumption of the BCF and BMF by using Equations (16) and (17). However, it is important to stress that although Equations (16) and (17) correct some potentially large errors in the calculation of the BCF and BMF of chemicals, they may not fully capture the effect of growth on the BCF and BMF. This is because respiration, feeding, excretion, growth, and metabolic transformation are closely related and interdependent; and their effect on chemical uptake and depuration remains an area requiring further investigation. However, given the current state of knowledge, it is useful to recognize that the strength of the BCF and BMF as metrics of bioaccumulation is that they represent a balance of uptake and depuration processes. Although the rates of uptake and depuration processes may change depending on external conditions (including the availability of food), the balance of mass and the corresponding ratio of uptake and depuration rates will generally be maintained over time. This makes the BCF and BMF fairly robust metrics of bioaccumulation under a variety of conditions, including growth and nongrowth. However, for the BCF and BMF to be useful metrics of bioaccumulation, it is important to ensure that in the calculation of the BCF and BMF mass balance is maintained.

## METHODS

### Relationship between feeding rate and growth rate

To investigate the relationship between the feeding rate and the growth rate in dietary bioaccumulation tests and test whether it is appropriate to use the feeding rate of a growing fish to represent the feeding rate of a nongrowing fish in the BMF calculation, we compiled reported feeding rates (*F*_*D*_) and growth dilution rate constants (*k*_g_) in OECD‐305‐style dietary bioaccumulation tests in juvenile rainbow trout (*Oncorhynchus mykiss*) reported in Lo et al. (2016).

### Relationship between respiration rate and growth rate

To explore the relationship between the respiration rate and the growth rate and test whether it is appropriate to use the respiration rate of a growing fish to represent the respiration rate of a nongrowing fish in BCF calculations, we followed the analysis of Jobling (1981), who recalculated data by Brett et al. (1969) and Brett (1976, 1979) to examine relationships between temperature, growth, and metabolism in juvenile sockeye salmon (*Oncorhynchus nerka*). Our modification to the analysis of Jobling (1981) included plotting the total rate of oxygen consumption (including the rates of oxygen consumption required for maintenance and growth) as a function of the fish's specific growth rate, which is the same as *k*_g_ in the present study. Maintenance is defined as the condition of the fish associated with a lack of growth. This modification makes it possible to determine the change in the fish's respiration rate attributable to growth. Because the rate of oxygen consumption at maintenance is a function of temperature, we determined the relationship between the rate of maintenance oxygen consumption in juvenile sockeye salmon, determined by Brett et al. (1969) and Brett (1976, 1979), and temperature. The rate of oxygen consumption at 12 °C, which is the temperature often used in bioaccumulation experiments with cold‐water species, was then determined from this relationship. Finally, we added the rate of oxygen consumption in the fish at maintenance and the rate of oxygen consumption associated with fish growth as determined by Jobling (1981).

### Growth correction in dietary bioaccumulation tests

To investigate methods for growth‐correcting the BMF, we applied the OECD‐305 growth‐correction method and the present study's proposed growth‐correction method (i.e., Equation (17)) to results from dietary bioaccumulation tests of octamethylcyclotetrasiloxane (D4) and decamethylcyclopentasiloxane (D5) in rainbow trout (*O. mykiss*) by Woodburn et al. (2013). In the study of Woodburn et al. (2013), 5‐g rainbow trout were fed in separate experiments food that contained D4 and D5 at concentrations of 457 ± 19.4 (standard deviation [SD]) and 458 ± 5.8 (SD) mg kg^–1^ at a rate of 0.03 kg food kg fish^–1^ d^–1^ for 35 d while being exposed to clean water without detectable concentrations of D4 and D5. After 35 d of exposure, fish were fed clean food to investigate the rate of depuration of D4 and D5. Throughout the experiments, fish were growing. The authors reported growth rate constants (*k*_g_) in the D4 experiment of 0.0383 d^–1^ during the uptake phase and 0.0279 d^–1^ during the depuration phase. Corresponding growth rate constants (*k*_g_) in the D5 experiment were 0.0351 d^–1^ during the uptake phase and 0.0264 d^–1^ during the depuration phase. The authors further reported depuration rate constants (*k*_T_) of 0.035 d^–1^ for D4 and 0.040 d^–1^ for D5 and dietary uptake efficiencies of 40% for D4 and 44% for D5. The time‐weighted mean lipid content of the fish in the D4 experiment was 6.32% ( ± 1.52 SD) and 5.64% ( ± 1.50 SD) in the D5 experiment. The mean lipid content of the fish food was 14.8% ( ± 0.1 SD).

### Growth correction in aqueous bioaccumulation tests

To investigate methods for growth‐correcting the BCF, we applied the OECD‐305 growth‐correction method and the present study's proposed growth‐correction method (i.e., Equation (16)) to results reported in Crookes and Brooke (2011) and in the guidance document to OECD‐305 (Organisation for Economic Co‐operation and Development 2017) from a bioconcentration experiment for a substance with a log *K*_OW_ of approximately 7 in rainbow trout (*O. mykiss*). The experiment consisted of a 35‐d uptake period, followed by a 42‐d depuration period. The mean measured exposure concentration was 0.34 μg L^–1^. Uptake and depuration rate constants were determined as 395 L kg^–1^ d^–1^ and 0.0432 d^–1^, respectively, and as 309 L kg^–1^ d^–1^ and 0.0382 d^–1^, respectively, using a second method described in Crookes and Brooke (2011). The growth dilution rate constant was 0.0298 d^–1^.

### Growth‐correction error in bioaccumulation tests

To explore the magnitude of potential errors in the determination of the BCF and BMF in bioaccumulation assessments attributable to mass‐balance violation, we calculated the BCFs and BMFs for nongrowing fish using the OECD‐305‐recommended method and Equations (16) and (17). The differences between the bioaccumulation metrics determined following the OECD‐305‐recommended method and the method described in the present study were considered to be errors in the determination of the BCF and BMF attributable to violation of mass balance. To do this, we analyzed the results from dietary bioaccumulation tests of 85 chemicals in juvenile rainbow trout using the methods recommended in OECD‐305 to determine *k*_T_ and *E*_*D*_ and reported feeding rates and growth dilution rate constants. We then compared BCFs and BMFs in nongrowing fish calculated by the OECD‐305‐recommended method and Equations (16) and (17).

## RESULTS AND DISCUSSION

### Relationship between feeding rate and growth rate

**Figure 1:**
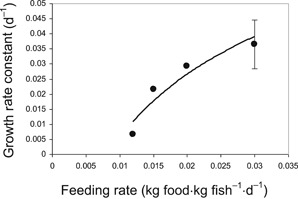
Measured growth rate constants in juvenile rainbow trout (*Oncorhynchus mykiss*) in Organisation for Economic Co‐operation and Development guideline 305–style dietary bioaccumulation tests as a function of the feeding rate in the test. Error bar represents the standard deviation.

Figure 1 illustrates that the growth rate increases with increasing feeding rate. A simple logarithmic relationship provides a good fit of the relationship between the growth rate constant (*k*_g_, per day) and the proportional feeding rate (*F*_*D*_, kilograms of food per kilogram of fish per day) in dietary bioaccumulation tests with juvenile rainbow trout:

(18) $$\begin{array}{l} k_{g}=0.0309\;(\pm 0.0069\;\text{SE})\;\cdot \;\ln\left(F_{D}\right)+0.147\;(\pm 0.028\;\left[SE\right])\\ \;r^{2}=0.91,n=4,p=0.047 \end{array}$$

where SE is the standard error. Similar relationships have been observed in a number of fish species (e.g., Kiørboe et al. 1987). These relationships illustrate that a lack of fish growth is associated with low feeding rates. It is possible to estimate at what feeding rate the growth dilution rate constant is expected to be zero in dietary bioaccumulation experiments with juvenile rainbow trout by solving Equation (18) for a *k*_g_ = 0, resulting in a feeding rate (*F*_*D*_) of 0.0085 kg food kg fish^–1^ d^–1^. This feeding rate may be useful in the calculation of a BMF for nongrowing fish. Figure 1 illustrates that the feeding rate in growing fish is substantially greater than the feeding rate in the same fish that do not grow. Hence, it is not correct to use the feeding rate of a growing fish to represent the feeding rate of a nongrowing fish. When calculating the BMF of a substance in a nongrowing fish by using the feeding rate of a growing fish, the BMF can be expected to be overestimated.

### Relationship between respiration rate and growth rate

Supplemental Data, Figure S1 illustrates that the rate of oxygen consumption O_2_ (milligrams of O_2_ per gram of fish per day) required for maintenance in juvenile sockeye salmon follows an exponential relationship with temperature (*T*, in degrees Celsius) that can be described by the following equation:

(19) $$\begin{array}{l} \;\ln\;O_{2}=0.093\;(\pm 0.0021\;\text{SE})\;\cdot \;T\;–\;0.118(\pm 0.034\;\text{SE})\\ \;n=5,r^{2}=0.9984,p<0.0001 \end{array}$$

Using Equation (19), the oxygen consumption rate required for maintenance in juvenile sockeye salmon at 12 °C can be estimated to be 2.71 mg O_2_ g fish^–1^ d^–1^. After adding the oxygen consumption rate at maintenance (i.e., no growth) and the oxygen consumption rate attributable to specific growth (Jobling 1981), the following relationship between the total oxygen consumption rate (O_2_) and specific growth rate (*k*_g_) is found:

(20) $$\begin{array}{l} O_{2}=353\;(\pm 77\;\text{SE})\;\cdot \;k_{g}+2.85\;(\pm 0.89\;\text{SE})\\ \;n=5,r^{2}=0.874,p=0.02 \end{array}$$

**Figure 2:**
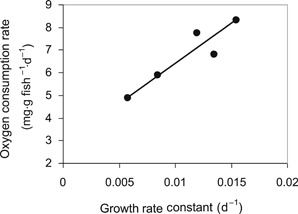
Oxygen consumption rates in juvenile sockeye salmon (*Oncorhynchus nerka*) as a function of the specific growth rate expressed in terms of the growth rate constant *k*_g_.

Figure 2, which illustrates this relationship, indicates that when fish grow, they respire more oxygen than when they do not grow. This relationship suggests that at a typical specific growth rate (*k*_g_) of 0.03 d^–1^, that is, similar to that measured by Woodburn et al. (2013) in juvenile rainbow trout, the oxygen consumption rate (O_2_) can be 4.7‐fold greater than that at a *k*_g_ of 0 d^–1^. In other words, the respiration rate in nongrowing juvenile fish is approximately 21% of that of growing fish. This illustrates that by using the respiration rate of a growing fish for the calculation of the BCF of a nongrowing fish, the BCF can be expected to be overestimated by a considerable amount.

### Growth correction of the BMF

Based on the reported observations by Woodburn et al. (2013), it is possible to determine the BMF of D4 and D5 as, respectively,

(21) $$\text{BMF}=\frac{E_{D}\;\cdot \;F_{D}}{k_{T}}=\frac{0.40\;\cdot \;0.03}{0.035}=0.34$$

(22) $$\text{BMF}=\frac{E_{D}\;\cdot \;F_{D}}{k_{T}}=\frac{0.44\;\cdot \;0.03}{0.040}=0.33$$

The corresponding growth‐corrected BMFs (BMF_NG_) of D4 and D5 as calculated by the OECD‐305‐recommended growth‐correction method are, respectively,

(23) $${\text{BMF}}_{\text{NG}}=\frac{E_{D}\;\cdot \;F_{D}}{k_{T}-k_{g}}=\frac{0.40\;\cdot \;0.03}{0.035-0.0279}=1.7$$

(24) $${\text{BMF}}_{\text{NG}}=\frac{E_{D}\;\cdot \;F_{D}}{k_{T}-k_{g}}=\frac{0.44\;\cdot \;0.03}{0.040-0.0264}=0.97$$

Equations (21) to (24) illustrate that the growth‐corrected BMFs are approximately 3 to 5 times greater than the actual BMFs. This large increase in the apparent BMF as a result of the growth correction is attributable to the use of the feeding rate for a growing fish (i.e., 0.03 kg food kg fish^–1^ d^–1^) in the numerator of BMF_NG_ but the depuration rate of a nongrowing fish. Figure 1 suggests that the feeding rate producing no growth in juvenile rainbow trout is approximately 0.0085 kg food kg fish^–1^ d^–1^ and 3.5 times lower than the feeding rate 0.03 kg food kg fish^–1^ d^–1^ used in the calculation. In the case of D4 and D5, the growth corrections cause substantial error, equivalent to the difference between the feeding rates of growing and nongrowing fish.

If both the uptake and depuration terms (i.e., numerator and denominator) are growth‐corrected as described by Equation (17), then the BMF_NG_ values of D4 and D5 are, respectively,

(25) $${\text{BMF}}_{\text{NG}}=\frac{E_{D}\;\cdot \;F_{D}}{\left(k_{T}-k_{g}\right)}\;\cdot \;\left(1-\frac{k_{g}}{k_{T}}\right)=\frac{0.40\;\cdot \;0.03}{\left(0.035-0.0279\right)}\cdot \;\left(1-\frac{0.0279}{0.035}\right)=0.34$$

(26) $${\text{BMF}}_{\text{NG}}=\frac{E_{D}\;\cdot \;F_{D}}{\left(k_{T}-k_{g}\right)}\;\cdot \;\left(1-\frac{k_{g}}{k_{T}}\right)=\frac{0.44\;\cdot \;0.03}{\left(0.040-0.0264\right)}\cdot \;\left(1-\frac{0.0264}{0.040}\right)=0.33$$

and essentially equal to the BMFs of D4 and D5 for growing fish.The term $\left(1-\frac{k_{g}}{k_{T}}\right)$, which is 0.20 in the experiment with D4 and 0.34 in the experiment with D5, is the fraction of the actual feeding rate that results in no growth, assuming that all other conditions of the fish remain the same. This suggests that the feeding rate expected to produce no significant growth was approximately 0.20 · 0.03 = 0.0060 kg food kg fish^–1^ d^–1^ in the experiment with D4 and 0.34 × 0.03 = 0.010 kg food kg fish^–1^ d^–1^ in the experiment with D5. These rates are in reasonable agreement with the feeding rate of 0.0085 kg food kg fish^–1^ d^–1^, calculated earlier for nongrowing juvenile rainbow trout in OECD‐305‐style dietary bioaccumulation tests. This suggests that the increase in the apparent BMF of D4 and D5 as a result of growth correction can be attributed to the selection of an inappropriate feeding rate in the BMF calculation for nongrowing fish.

The lipid‐normalized BMFs (BMF_L_) of D4 and D5 derived from the results of the dietary bioaccumulation tests are 0.34 × (0.148/0.0632) or 0.80 kg lipid kg lipid^–1^ for D4 and 0.33 × (0.148/0.0564) or 0.87 kg lipid kg lipid^–1^ for D5 in growing and nongrowing fish. These BMFs are in reasonable agreement with observed trophic magnification factors of D4 and D5 in aquatic food webs, which range between 0.31 and 1.3 kg lipid kg lipid^–1^ for D4 and between 0.18 and 2.3 kg lipid kg lipid^–1^ for D5 (Powell et al. 2009, 2017, 2018; Borga et al. 2012, 2013; McGoldrick et al. 2014; Jia et al. 2015). In contrast, corresponding BMFs calculated with the OECD‐305 growth correction are 4.0 and 2.5 kg lipid kg lipid^–1^ and are greater than observed trophic magnification factors of D4 and D5 in aquatic ecosystems. This suggests that BMFs determined in dietary bioaccumulation tests can be adequate descriptors of biomagnification in the environment as long as mass balance is adhered to in the extrapolation of bioaccumulation data from growing to nongrowing fish.

### Growth correction of the BCF

Following the example in Crookes and Brooke (2011), the BCF of the substance can be calculated as

(27) $$\text{BCF}=\frac{k_{1}}{k_{T}}=\frac{395}{0.0432}=9144$$

or

(28) $$\text{BCF}=\frac{k_{1}}{k_{T}}=\frac{309}{0.0382}=8089$$

in liters per kilogram of fish depending on the method of calculation of *k*_1_ and *k*_T_. The corresponding growth‐corrected BCF_NG_ values are, respectively,

(29) $${\text{BCF}}_{\text{NG}}=\frac{k_{1}}{k_{T}-k_{g}}=\frac{395}{0.0432-0.0298}=29\;500$$

(30) $${\text{BCF}}_{\text{NG}}=\frac{k_{1}}{k_{T}-k_{g}}=\frac{309}{0.0382-0.0298}=36\;800$$

in liters per kilogram of fish. The growth‐corrected BCFs are approximately 3.2 to 4.5 times greater than the actual BCFs. This increase is attributable to representing the respiration rate constant (*k*_1_) for a nongrowing fish by the respiration rate constant of a growing fish. Equation (20) shows that at a growth rate 0.03 d^–1^, the respiration rate in juvenile *O. nerka* is approximately 4.7‐fold greater than that at a growth rate of 0 d^–1^. Hence, the increase in the BCF on growth correction can be attributed to the overestimation of the respiration rate by the growth‐correction method. This suggests that the OECD‐305‐recommended growth correction produces unrealistic estimates of the BCF because it uses a *k*_1_ for a growing fish, which is much greater than the *k*_1_ for a nongrowing fish. If both the uptake and depuration terms (i.e., numerator and denominator) are growth‐corrected as described by Equation (16), then the BCF_NG_ is

(31) $${\text{BCF}}_{\text{NG}}=\frac{k_{1}}{\left(k_{T}-k_{g}\right)}\;\cdot \;\left(1-\frac{k_{g}}{k_{T}}\right)=\frac{395}{\left(0.0432-0.0298\right)}\;\cdot \;\left(1-\frac{0.0298}{0.0432}\right)=9144$$

or

(32) $${\text{BCF}}_{\text{NG}}=\frac{k_{1}}{\left(k_{T}-k_{g}\right)}\;\cdot \;\left(1-\frac{k_{g}}{k_{T}}\right)=\frac{309}{\left(0.0382-0.0298\right)}\cdot \;\left(1-\frac{0.0298}{0.0382}\right)=8089$$

in liters per kilogram of fish, which are equal to the BCFs calculated in Equations (27) and (28). In this case, the term $\left(1-\frac{k_{g}}{k_{T}}\right)$, which is 0.31 or 0.22 in this particular test, represents the fraction of the respiration rate associated with no growth. This fraction is in line with the earlier observation that in juvenile sockeye salmon respiration rates in nongrowing fish are approximately 21% (or 0.21) of the respiration rates in fish growing at a rate of 0.03 d^–1^. This supports the use of Equation (16) for estimating BCFs of chemicals in fish that do not grow.

### Growth‐correction error in bioaccumulation tests

**Figure 3:**
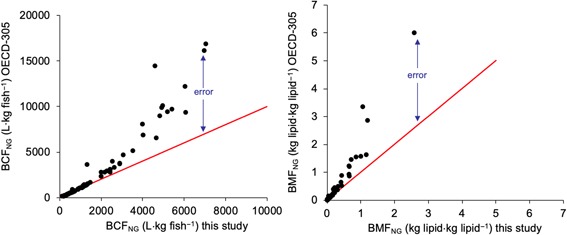
Growth‐corrected bioconcentration factors (left) for fish with a 5% lipid content and growth‐corrected biomagnification factors (right) determined from the results of dietary bioaccumulation tests according to equations (16) and (17) in the present study (*x*‐axis) and by the growth‐correction method in Organisation for Economic Co‐operation and Development guideline 305 (OECD‐305; *y*‐axis). The red line is the 1:1 line. The difference between the black dots and the red line represents the magnitude of error attributable to violation of mass balance in the OECD‐305 growth‐correction method. BCF = bioconcentration factor; BMF = biomagnification factor; NG = nongrowing.

Figure 3 illustrates the error caused by the growth correction for substances with varying BCFs and BMFs. Figure 3 shows that the growth‐correction error is small and inconsequential for substances that exhibit low BCFs and BMFs. This is because depuration rate constants (*k*_E_) are much greater than the growth rate constants (*k*_g_). However, if BCFs are close to 5000 L kg fish^–1^ and/or BMF_L_ values are close to 1 kg lipid kg lipid^–1^, then the error becomes significant and reaches levels up to 300%. For these substances, depuration rates are relatively small and growth dilution has a significant effect on the BCFs and BMFs. The magnitude of the error is large enough to cause misclassification of chemicals that exhibit BCFs and BMFs near key bioaccumulation criteria values.

### Recommendations for growth correction

The results of this analysis indicate that it is both incorrect and unnecessary to include a growth correction for assessing BCFs and BMFs in nongrowing fish. The current OECD‐305‐recommended methods for assessing BCFs and BMFs in growing fish are adequate for assessing BCFs and BMFs in nongrowing fish. This is because the BCF and BMF are fairly robust metrics of bioaccumulation because they represent a mass balance as a ratio of uptake rates (in the numerator) and related depuration rates (in the denominator). Any changes in the depuration rate are therefore often matched by corresponding changes in the uptake rate (and vice versa), leaving the ratio relatively unaffected. However, it is important to stress that relationships between feeding, respiration, growth, and metabolic transformation are highly complex and perhaps currently insufficiently understood to fully anticipate the effect of growth on bioconcentration and biomagnification. More research may be required to better comprehend the effect of growth on bioaccumulation. However, before this knowledge is obtained, it is best to rely on well‐established mass‐balance principles to guide assessments of bioaccumulation. A recent proposal (Hashizume et al. 2018) to normalize the BMF to a dietary lipid content of 5% is therefore best considered when applied to the actual BMF rather than the growth‐corrected BMF.

## Supplemental Data

The Supplemental Data are available on the Wiley Online Library at DOI: 10.1002/etc.4509.

## Data Accessibility

Data, associated metadata, and calculation tools are available from the corresponding author (gobas@sfu.ca).

## Supporting information

This article includes online‐only Supplemental Data.

Supporting information
