# Supplementary material for: Growth‐Correcting the Bioconcentration Factor and Biomagnification Factor in Bioaccumulation Assessments
Source: Environ Toxicol Chem. 2019 Aug 1;38(9):2065–72. doi: 10.1002/etc.4509 (PMC6852268; doi:10.1002/etc.4509)
Supplement: Supplementary file 1 — Supporting information [file ETC-38-2065-s001.docx]

**SUPPORTING INFORMATION**


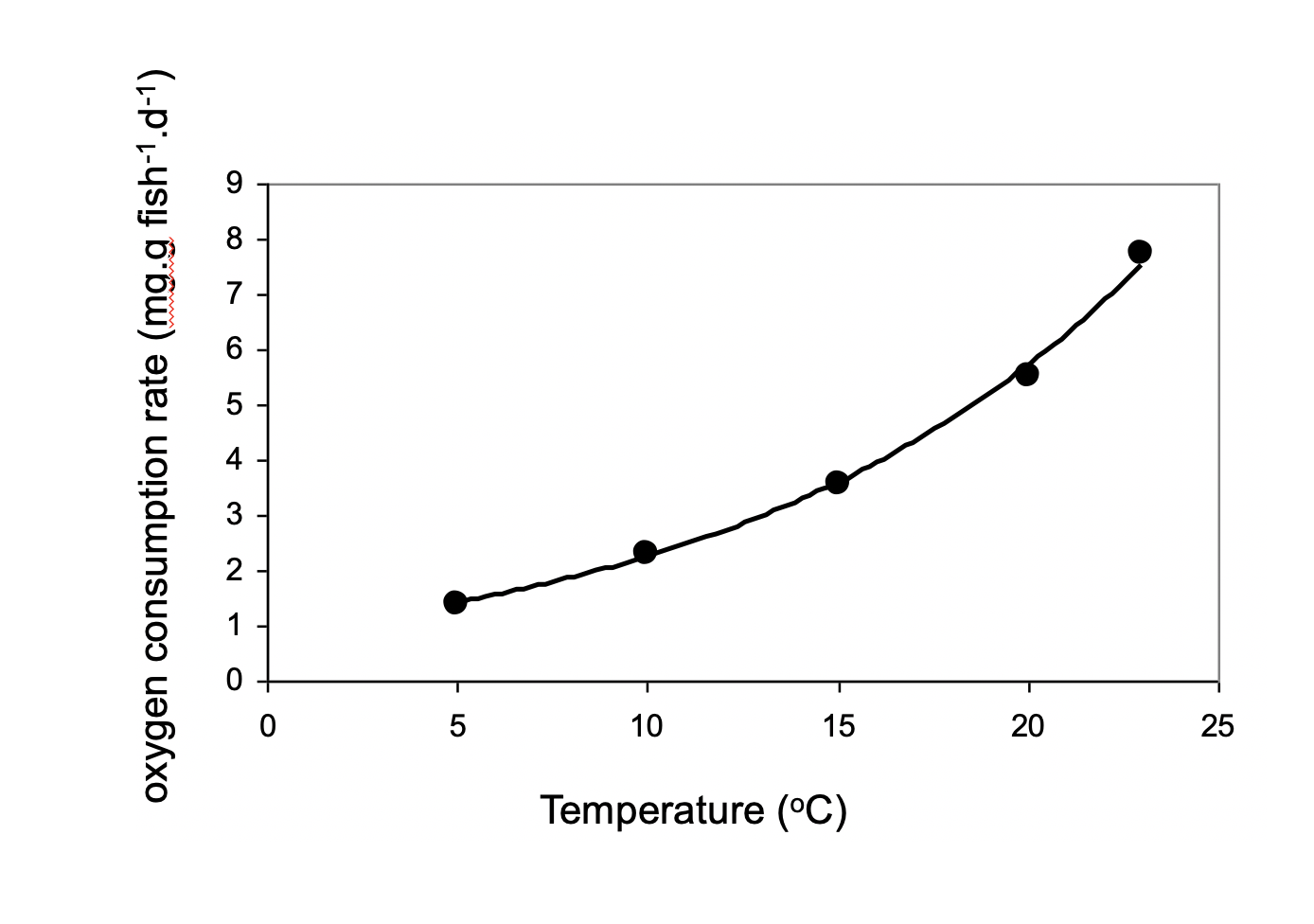


Figure S1: Oxygen consumption rates (in units of mg O_2_.g fish^-1^.d^-1^ ) in juvenile sockeye salmon (Oncorhynchus nerka) as a function of temperature (in units of ^o^C).

**Step-by-step Derivations of equations 14 and 15:**

Equation 14:

${k_{1,NG}\cdot C_{W}=k}_{1}\cdot C_{W}-k_{g}\cdot C_{F}$ (S1)

Divide by C_W_:

${k_{1,NG}=k}_{1}-k_{g}\cdot\frac{C_{F}}{C_{W}}$ (S2)

Substitute equation 11 into S2.

${k_{1,NG}=k}_{1}-k_{g}\cdot\frac{k_{1}}{k_{T}}$ (S3)

or

$k_{1,NG}=k_{1}\cdot(1-\frac{k_{g}}{k_{T}})$ (S4)

Equation 15

$E_{D}\cdot F_{D,NG}\cdot C_{D}{=E}_{D}\cdot F_{D}\cdot C_{D}-k_{g}\cdot C_{F}$ (S5)

Divide by E_D_.C_D_:

${F_{D,NG}=(E}_{D}\cdot F_{D}\cdot C_{D}-k_{g}\cdot C_{F})/{(E}_{D}\cdot C_{D})=F_{D}-\frac{k_{g}}{E_{D}}\cdot\frac{C_{F}}{C_{D}}$ (S6)

Substitute equation 11 , i.e.,

$\frac{C_{F}}{C_{D}}= \frac{{E_{D}\cdot F}_{D}}{k_{T}}$ (S7)

into S6, giving:

${F_{D,NG}=(E}_{D}\cdot F_{D}\cdot C_{D}-k_{g}\cdot C_{F})/{(E}_{D}\cdot C_{D})=F_{D}-\frac{k_{g}}{E_{D}}\cdot\frac{{E_{D}\cdot F}_{D}}{k_{T}}$ (S8)

or

${F_{D,NG}=(E}_{D}\cdot F_{D}\cdot C_{D}-k_{g}\cdot C_{F})/{(E}_{D}\cdot C_{D})=F_{D}\cdot(1-\frac{k_{g}}{k_{T}})$ (S9)
